# Deodorization of noni juice using rotary evaporation and its sensory and chemical properties in pineapple juice blends

**DOI:** 10.1038/s41598-025-18691-7

**Published:** 2025-10-07

**Authors:** Zahra Yusufali, Matthew Siderhurst, Beatrice Kim-Lee, Xiuxiu Sun

**Affiliations:** 1 United States Department of Agriculture, Agricultural Research Service, Daniel K. Inouye U.S. Pacific Basin Agricultural Research Center, 64 Nowelo Street, Hilo, HI 96720 USA; 2 Pomona College, 333 N College Way, Claremont, CA 91711 USA; 3 United States Department of Agriculture, Agricultural Research Service, USDA Southeastern Fruit and Tree Nut Research Station, 21 Dunbar Road, Byron, GA 31008 USA

**Keywords:** *Morinda citrifolia*, Rotary evaporation, Flavor, Taste, Gas chromatography–mass spectrometry, Principal component analysis, Mass spectrometry, Cheminformatics

## Abstract

Juice from noni fruit, *Morinda citrifolia*, has multiple health benefits but the unpleasant odors of raw juice limits consumer adoption. The two-step process of deodorizing noni juice by rotary evaporation (rotovap) followed by mixing with pineapple juice produces a beverage that has a less off-putting odor and is more palatable. Analysis of total soluble solids and titratable acidity confirms that pineapple juice sweetens the taste profile. Sensory evaluation by trained panelists found that 80% pineapple with 20% rotovap (20 RVN) received higher scores for both sweetness and freshness than 80% pineapple and 20% original noni juice (20 OGN) highlighting the impact of noni juice volatiles on these organoleptic qualities. Noni juice volatiles were further investigated using dynamic headspace sampling (HS) coupled to gas chromatography/mass spectrometry (GC-MS) with principal component analysis (PCA) of volatile profiles. These analyses show that total volatiles decreased in rotovap treated juices, pineapple juice and pineapple/noni juice mixtures grouped more closely while untreated (OGN) and the water fraction removed by rotovap were strongly associated. Together these results suggest that treatment by rotovap removes unpleasant odors from noni juice and mixture with pineapple juice produces a sweeter, fresher, more palatable beverage potentially suitable for the consumer market.

## Introduction

Noni (*Morinda citrifolia* L.) is a small evergreen tree or shrub native to South Asia, that grows abundantly in the tropics. It has long been used as a medicinal plant in Polynesia, South Asia, and Northeastern Australia^1^and was one of the canoe plants that were brought to the Hawaiian Islands by the Polynesians^2^. Thus, noni has high cultural significance in Hawaii, having been used by ancient Hawaiians as a traditional medicine to heal broken bones, ease asthma, and reduce diabetes, hypertension, digestive disorders, and menstrual cramps^1^. Due to its traditional medicinal uses around the world, numerous studies have been done in search of evidence for noni’s presumed nutritional benefits. In vivo studies have found that noni juice improves joint health, increases physical endurance, increases immune activity, aids weight management, helps maintain bone health and normal blood pressure, and improves gum health^3^. Numerous in vitro studies have also shown that noni exhibits antibacterial, antifungal, antiviral, antioxidant, anti-inflammatory, cardiovascular, anti-cancer, and hypotensive activity, as well as many other health benefits^4^.

Noni has gained increased consumer appeal in recent years, due to research and advertising praising it as a functional food. In 2022, the global noni juice market was valued at 66 million US dollars and is forecasted to grow to 109.4 million US dollars by 2029 ^5^. In 2021, noni was the second highest-valued fruit crop grown in Hawaii at USD 1.99 million^6^. Noni fruit is rich in compounds such as vitamin C, potassium, iridoid glucosides, americanin A, neoligan, among others. Americanin A and neolignan have been isolated and found in in vivo studies to be potent antioxidants^7^. Due to its high nutritional and medicinal value, noni has been used to produce consumer functional foods such as capsules, juices, or teas^4^. However, noni juice is known to have a sour, astringent flavor that significantly limits its marketability at higher concentrations^2^. Noni’s unfavorable flavor is conventionally masked through fermentation, which, at the factory-level, decreases the concentration of octanoic acid and hexanoic acid, two main contributors to the pungent odor of noni juice^8^.

Pineapple (*Ananas comosus*), on the other hand, is highly palatable and commercialized in Hawaii, with nearly 100% of the 168,020 metric tons of pineapples produced in the United States in 2021 being from Hawaii^9^. In recent years, pineapple juice has been blended with other less palatable but functional fruit juices such as turmeric and ginger to enhance its nutritional capabilities and increase palatability^10–12^. Deodorization techniques such as rotary evaporation and deacidification have also been used as techniques to improve the scent of nutritious foods by removing volatile compounds responsible for pungent odors^13,14^.

The purpose of this study was to develop a healthy and marketable juice by combining the nutritional benefits of noni with the palatability of pineapple juice and then evaluating the physical, chemical, and sensory properties of the juice. The methodology was to first use rotary evaporation to remove volatile compounds responsible for the pungent odor of noni juice, then blend the evaporated noni juice with pineapple juice to combine the nutritional benefits of noni juice with the appealing taste of pineapple juice. This research has the potential to make a positive impact on Hawaii, as it takes advantage of two of the most abundant and commercialized fruits in the state. In addition, the development and production of a fortified drink using noni raises awareness about the health benefits of noni and its cultural significance within Hawaii, presenting the otherwise niche product to a wider consumer market. Finally, the increased cultivation of noni in Hawaii may reduce overdependence on food imports from other states and nations.

## Materials and methods

### Materials and juice Preparation

Pineapples were purchased from a local store and juiced using a cold press juicer (PURE Juicer Company, Seattle, WA, USA). The juice was pasteurized at 71 °C for 30s. 100% natural fermented noni juice was purchased online (Noni Liquids LLC, Pahoa, HI, USA). The deodorized noni juice was made using a rotary evaporator (R-300, BUCHI Corporation, New Castle, DE, USA). The rotary evaporator water bath was set to 35 °C and the rotation of the round bottom flask was set to 100 rpm. To evaporate the water along with the volatiles, the pressure was gradually reduced to 20 mbar.

Initially, the °Brix value of the original noni juice (from commercial bottle) was measured at 6.8%. Subsequently, 100 mL of this juice was concentrated using rotary evaporation at 35 °C and 20 mbar for approximately one hour, yielding a final volume of about 5 mL and a °Brix value of about 70%. Deionized water was then added back to the concentrated juice to restore its °Brix value to that of the original juice. The juice blends are listed in Table 1.

**Table 1:** The juice samples and abbreviation for pineapple and noni juice.

Juice	Abbreviation
Original noni juice	OGN
Rotary evaporated noni juice	RVN
Rotovap water distillate	Fraction
100% pineapple juice (control)	100 P
90% pineapple juice, 10% original noni juice	10 OGN
90% pineapple juice, 10% rotovap noni juice	10 RVN
80% pineapple juice, 20% original noni juice	20 OGN
80% pineapple juice, 20% rotovap noni juice	20 RVN

### Total soluble solids (TSS), titratable acidity (TA), and pH

The total soluble solids (TSS), titratable acidity (TA), and pH were measured using a digital refractometer (PAL-3, ATAGO U.S.A., Inc., Bellevue, WA, USA), a fruit acidity meter (GMK-835 F, G-WON, Korea), and a pH meter (Accumet AB315, Thermo Fisher Scientific, Waltham, MA USA), respectively. The results for the TSS were expressed as °Brix and the TA as %. In addition, a TSS/TA ratio for each juice sample was calculated using their respective measurements.

### Volatiles

The volatile compounds in the juice blends (Table 1) were analyzed using static headspace gas chromatography-mass spectrometry (HS-GC–MS) following methods from our previous research^11,12^. To prepare the samples, 1 g of NaCl was combined with 3 mL of juice sample into a 20 mL headspace vial sealed with a silicone septa crimp cap. The vial was incubated in the headspace autosampler (Agilent 7697 A, Agilent Technologies, Santa Clara, CA) oven at 45 °C for 10 min with agitation at 18 shakes/min and an acceleration of 60 cm/s². Nitrogen carrier gas was used to fill the vial to a pressure of 15 psi, facilitating the transfer of headspace volatiles into the sample loop. The sample loop temperature was maintained at 120 °C. The headspace volatiles were then injected into the GC-MS (Agilent 8890, Agilent Technologies, Santa Clara, CA) over a duration of 0.50 min, with a split ratio of 20:1.

The GC was equipped with a HP-5ms 5% Phe column (30 m × 0.25 mm i.d., 0.25 μm film thickness, Agilent, Santa Clara, CA) and helium was used as a carrier gas. The oven temperature was initially held at 40 °C for 2 min, then ramped up to 280 °C at 10 °C/min. The MS transfer line and ion source temperatures were set to 290 °C and 250 °C, respectively. Mass spectrometric data was collected with electron ionization (70 eV) over a fragment range of 45–350 *m/z*. Compounds were identified by comparing their mass spectra with entries in the NIST library. Additionally, for each compound, Linear Retention Indices (LRI) were calculated using a standard mixture of saturated C7–C40 alkanes (each component at 1 mg/ mL in hexane, certified reference material) and compared to literature reported retention indices.

### Sensory evaluation

Ten trained panelists evaluated four juices (10 OGN, 10 RVN, 20 OGN, and 20 RVN) and 100% pineapple juice as a control. They assessed the aroma attributes of grassy, cooked vegetables, earthy, fresh, and cheesy, and taste and mouthfeel attributes of sweet, sour, bitter, fermented, and astringent. Ratings were recorded on a 0–10 intensity scale, where 0 represented no perception and 10 represented the highest intensity (Yusufali et al. 2024). Each panelist received 10 mL of each juice in 50 mL plastic cups with lids (SOLO, Urbana, IL). Panelists were instructed to cleanse their palates between samples using water.

### Statistical analysis

Principal Component Analysis (PCA) was performed using ClustVis^15^ on the full chromatogram data as described by Siderhurst et al.^16^which included all recorded total ion chromatograms (TIC) for each juice sample and the water fraction in triplicates. Preliminary analysis showed that only data up to the 13.00-min retention time was relevant, as no compounds were detected after that point, so all traces were trimmed at 13 min. TIC data was analyzed in two ways, (1) TIC data standardized by run was subjected to PCA to identify profile differences, and (2) unstandardized (untransformed) TIC data was subjected to PCA to examine variation based on the relative abundance of each compound in the juice samples and water fraction. Standardization by run was accomplished using the ‘standardize’ function in Excel. Loading plot data for each PCA was prepared using Excel by plotting both principal components 1 and 2 (y-values) against retention time (x-axis) for both standardized and unstandardized PCAs.

One-way analysis of variance (ANOVA) with Tukey’s HSD post-hoc tests were performed for each of the following groups: TSS, TA, TSS/TA, pH, and volatiles with the independent factor of each juice sample. Each ANOVA was performed using JMP statistical analysis software (version 17; SAS Institute, Cary, NC). All statistical analyses were conducted in triplicates with a significance level set at α ≤ 0.05.

## Results and discussion

### Total soluble solids (TSS), titratable acidity (TA), and pH

**Fig. 1:**
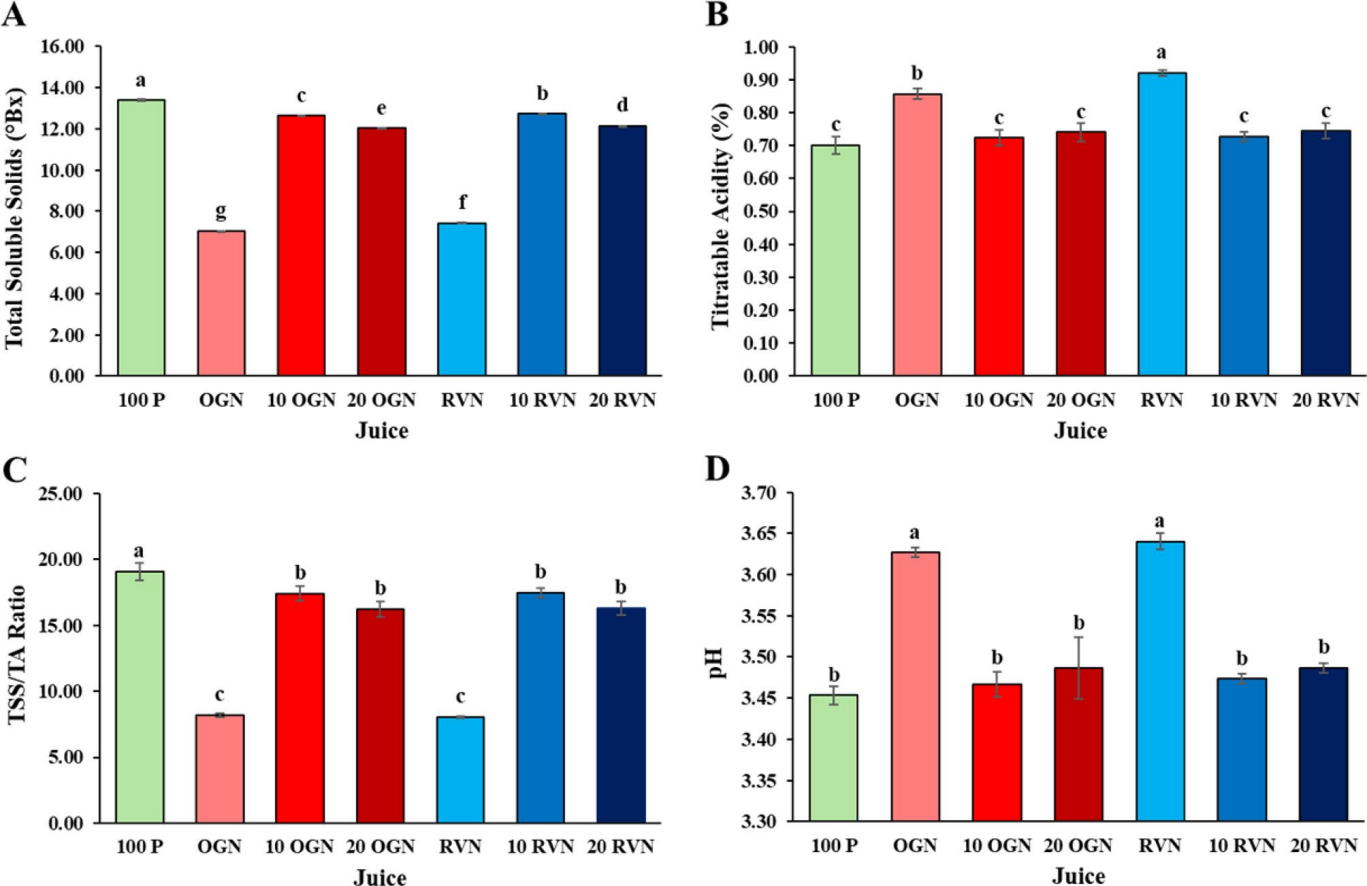
Mean values for **(A)** total soluble solids (°Brix), **(B)** titratable acidity (%), **(C)** TSS/TA ration, and **(D)** pH for each juice sample. Vertical bars show standard deviations; letters denote significant differences (ANOVA, Tukey’s HSD, *p* < 0.05).

The data indicates that 100% pineapple juice has the highest total soluble solids, making it the sweetest sample, while OGN has the lowest, indicating the least sweetness (Fig. 1). The addition of noni juice slightly lowers the sweetness of juice blends as indicated by decreasing TSS values. For Titratable Acidity (TA), RVN has the highest acidity, while 100 P has the lowest. The TSS/TA ratio is notably higher in 100 P, indicating a sweeter taste profile, whereas RVN and OGN have lower ratios, indicating the inverse. When looking at pH, RVN and OGN exhibit higher values compared to 100 P, suggesting a less acidic composition.

### Volatiles

**Fig. 2:**
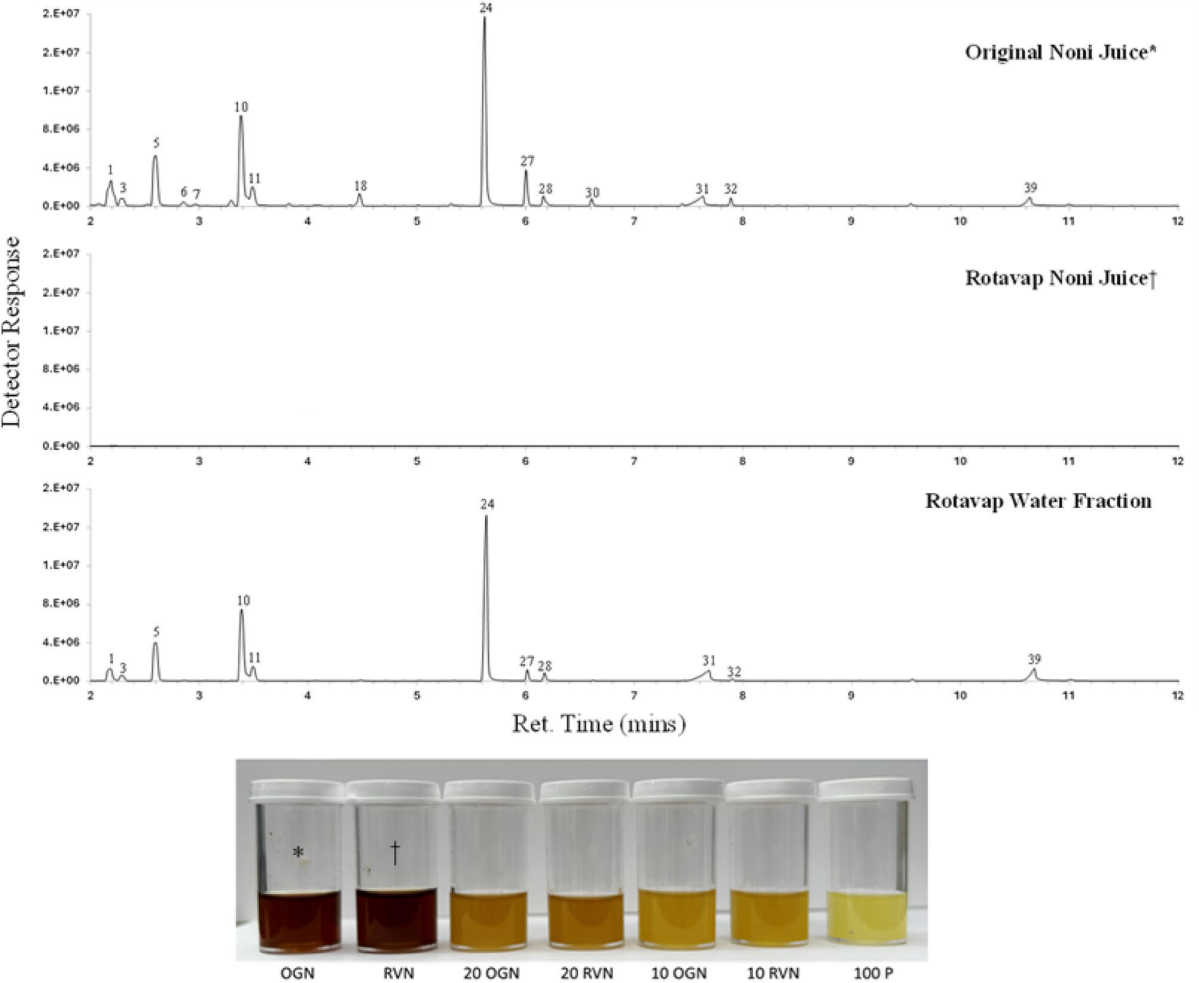
Example GC traces of original noni (*), rotovap noni (†), and water fraction, with juice samples for reference. All GC traces are shown at the same scale to simplify comparisons between the juices. Compounds were identified via retention indices, mass spectra (NIST library), and literature; peak numbers correspond to Table 2.

Altogether, 24 samples of juice were analyzed by headspace auto sampling coupled to GC-MS. Figure 2 shows example chromatograms of the original noni juice, rotary evaporated noni juice, and the resulting water fraction. The numbered peaks correspond to those listed in Table 2. Compounds which presumably contribute to the pungent or unpleasant smell in noni juice include hexanoic acid and octanoic acid, identified by numbers 31, and 39 respectively^8^. While fruity or pleasant volatiles are also present in noni juice, their aroma impact appears to be synergistically masked by the intensity of the pungent compounds. This reflects the concept of odor thresholds in foods, where dominant volatiles can overpower more subtle ones^17^. In the case of noni juice, the fruity volatiles simply do not have enough aromatic strength to overcome the pungent ones, resulting in an overall unpleasant odor profile. Note that all traces in Fig. 2 are shown at the same relative intensity, thus no compounds are readily apparent in the rotary evaporated noni juice; most volatiles moved into the vapor phase and were collected in the water distillate. From an instrumental perspective, this suggests that the rotary evaporation treatment may effectively deodorized the noni juice. While GC volatile analysis can identify and quantify volatile compounds, human perception depends on whether each compound’s concentration exceeds its odor threshold. Therefore, taste testing by human subjects are needed to evaluate whether people would be willing to consume the food.

**Table 2:** Volatile compounds identified through HS-GC-MS from noni juice, pineapple juice, and mixtures of the two juices (including Rotovap fractions).

Pk #	Ret. time	LRI (HP5-MS)	Compound	Descriptors	Average Abundance (total ion current × 10^7^)							
					100 *P*	10 OGN	10 RVN	20 OGN	20 RVN	OGN	RVN	Fraction
1	2.19	608	2-Methyl-3-buten-2-ol	Fruity	–	–	–	–	–	5.2 ± 2.3a	–	4.6 ± 0.1a
2	2.20	610	Ethyl acetate	Solvent-like, fruity	35.1 ± 0.2a	22.6 ± 0.1b	25.1 ± 2.5b	16.9 ± 0.2c	15.3 ± 0.3c	–	–	–
3	2.30	620	Isobutanol	Wine, solvent, bitter	–	0.3 ± 0.0b	–	0.4 ± 0.0b	–	1.7 ± 0.7ab	–	1.9 ± 0.0a
4	2.53	647	Isopentanal	Gasoline-like	–	–	–	–	–	0.2 ± 0.2a	–	–
5	2.60	656	1-Butanol	Fruity	–	1.1 ± 0.0b	–	2.1 ± 0.1b	–	10.1 ± 3.4a	–	12.6 ± 0.3a
6	2.86	686	2-Pentanone	Acetone like	–	0.0 ± 0.0c	–	0.1 ± 0.0c	–	1.3 ± 0.0a	–	0.2 ± 0.0b
7	2.98	700	Pentanal	Pungent, almond-like	–	–	–	–	–	0.7 ± 0.0a	–	0.1 ± 0.0b
8	3.19	715	1-Propyl acetate	Mild, fruity	0.7 ± 0.0a	0.4 ± 0.0b	0.4 ± 0.0b	0.3 ± 0.0c	0.2 ± 0.0c	–	–	–
9	3.30	722	Methyl butanoate	Fruity, sweet	0.6 ± 0.0a	0.4 ± 0.0b	0.3 ± 0.0bc	0.3 ± 0.0c	0.2 ± 0.0d	–	–	–
10	3.39	728	2-Methyl-1-buten-4-ol	Sweet, fruity	–	1.7 ± 0.0b	–	3.4 ± 0.1b	–	16.2 ± 5.3a	–	21.0 ± 0.5a
11	3.50	736	2-Methyl-1-butanol	Ripe fruit	–	0.3 ± 0.0d	–	0.7 ± 0.0d	–	5.8 ± 0.1a	–	4.3 ± 0.1b
12	3.83	759	Ethyl isobutanoate	Sweet, fruity	0.0 ± 0.0a	–	–	–	–	0.3 ± 0.2a	–	–
13	3.95	766	1-Pentanol	Pungent, alcohol-like	–	–	–	–	–	0.1 ± 0.1a	–	0.1 ± 0.0a
14	4.06	774	Isobutyl acetate	Fruit, apple, banana	0.2 ± 0.0a	0.1 ± 0.0b	0.1 ± 0.0b	0.1 ± 0.0c	0.0 ± 0.0d	–	–	–
15	4.07	775	Prenol	Fruity	–	–	–	–	–	0.1 ± 0.0a	–	0.1 ± 0.0a
16	4.11	778	Methyl 2-methylbutanoate	Fruity, pineapple	0.8 ± 0.0a	0.4 ± 0.0bc	0.4 ± 0.0b	0.3 ± 0.0 cd	0.2 ± 0.0d	–	–	–
17	4.25	787	Diethyl carbonate	Mild, fruity	0.1 ± 0.0a	0.0 ± 0.0b	0.0 ± 0.0bc	0.0 ± 0.0c	0.0 ± 0.0d	–	–	–
18	4.49	803	Ethyl butanoate	Sweet, fruity	0.8 ± 0.0b	0.5 ± 0.0c	0.4 ± 0.0d	0.3 ± 0.0e	0.2 ± 0.0f	3.0 ± 0.0a	–	0.3 ± 0.0ef
19	4.72	816	Butyl ethanoate	Fruity	–	–	–	–	–	0.1 ± 0.0a	–	–
20	5.03	834	Furfural	Sweet, bread, almond	–	–	–	–	–	0.1 ± 0.0a	–	0.1 ± 0.0a
21	5.33	852	Ethyl 2-methylbutanoate	Fruity	0.6 ± 0.0a	0.3 ± 0.0b	0.3 ± 0.0b	0.2 ± 0.0c	0.1 ± 0.0d	–	–	–
22	5.33	852	(E)-Hex-3-en-1-ol	Moss, fresh	–	–	–	–	–	0.3 ± 0.1a	–	0.2 ± 0.0a
23	5.38	855	(Z)-Hex-3-en-1-ol	Grass	–	–	–	–	–	0.1 ± 0.1a	–	0.1 ± 0.0a
24	5.63	869	1-Hexanol	Green	–	3.2 ± 0.0d	–	6.0 ± 0.1c	–	43.0 ± 0.3a	–	39.4 ± 0.6b
25	5.79	878	Isoamyl acetate	Banana	0.5 ± 0.0a	0.4 ± 0.0b	0.2 ± 0.0d	0.3 ± 0.0c	0.1 ± 0.0e	–	–	–
26	5.93	887	3-Methyl-3-buten-1-ol, acetate	Fruity	–	–	–	–	–	0.1 ± 0.1a	–	0.1 ± 0.0a
27	6.02	891	2-Heptanone	Soapy, fruity	–	0.2 ± 0.0a	–	0.3 ± 0.0a	–	3.4 ± 1.5a	–	2.0 ± 0.0a
28	6.18	901	2-Heptanol	Sweet, floral, fruity	–	0.1 ± 0.0c	–	0.2 ± 0.0c	–	2.3 ± 0.1a	–	1.7 ± 0.1b
29	6.21	903	Ethyl pentanoate	Fruity	0.0 ± 0.0a	–	–	–	–	–	–	–
30	6.62	926	Methyl hexanoate	Fruity, fresh, sweet	0.9 ± 0.0a	0.6 ± 0.0ab	0.1 ± 0.1b	0.4 ± 0.0ab	0.2 ± 0.0b	0.6 ± 0.3ab	–	0.1 ± 0.0b
31	7.59	982	Hexanoic acid	Rancid, pungent, unpleasant	–	–	–	–	–	5.7 ± 0.2b	–	8.1 ± 0.6a
32	7.90	1000	Ethyl hexanoate	Fruity	2.5 ± 0.0a	1.8 ± 0.0ab	0.4 ± 0.4d	1.2 ± 0.0bc	0.7 ± 0.1 cd	–	–	–
33	8.07	1010	(E)-Ethyl-3-hexenoate	Green, fruity	0.0 ± 0.0a	–	–	–	–	–	–	–
34	8.34	1027	Methyl 3-methylthiopropanoate	Sulfurous, fruity	–	0.0 ± 0.0b	–	0.0 ± 0.0b	–	0.1 ± 0.0a	–	–
35	9.07	1072	1-Octanol	Sweet, floral, fruity	–	–	–	–	–	0.2 ± 0.0a	–	0.1 ± 0.1a
36	9.56	1101	Linalool	Flowery	–	–	–	–	–	0.5 ± 0.0a	–	0.4 ± 0.0b
37	9.57	1102	Ethyl 3-methylthiopropanoate	Pineapple-like	0.0 ± 0.0c	0.1 ± 0.0b	0.0 ± 0.0c	0.1 ± 0.0a	0.0 ± 0.0c	–	–	–
38	9.93	1126	Methyl octanoate	Fruity	–	0.0 ± 0.0a	–	0.0 ± 0.0a	–	0.1 ± 0.0a	–	–
39	10.65	1172	Octanoic acid	Sweaty	–	–	–	0.0 ± 0.0c	–	4.3 ± 0.3b	–	6.5 ± 0.4a
40	11.00	1196	Ethyl octanoate	Fruity, fatty	–	0.1 ± 0.0b	–	0.1 ± 0.0b	–	0.6 ± 0.0a	–	0.5 ± 0.1a

The letters indicate significant differences in volatiles between the juice samples (ANOVA, Tukey’s HSD, *p* < 0.05).

The observed color of the juices (Fig. 2) shows that the rotary evaporated noni juice matched in color with the original noni juice (both dark in color), demonstrating only a slight observable change in color due to the deodorization process. The juice blends created with the rotary evaporated noni juice (Table 1), do not indicate an observable color difference compared to the blends containing the original noni juice, eliminating any potential bias during sensory evaluation due to color. Pineapple juice was noticeably lighter in color than all other juices and mixtures of juices had colors reflective of the percentage noni/pineapple.

A total of 40 volatiles were detected across pineapple juice, noni juice, and their blends, with average peak areas presented in Table 2. Among these, 28 volatiles were identified in noni juice, 23 of which were also found in the water fraction post-deodorization. Interestingly, three compounds (isopentanal, ethyl isobutanoate, and butyl ethanoate) were only found in the noni juice and not in the water fraction of rotavap juice. This may be due to the compounds being split between the fractions and therefore at low concentrations which might fall below the GC-MS detection threshold. It is also possible that these three compounds are somewhat less stable and either degrade or undergo transformations with heating although neither of the esters nor the aldehyde are widely reported as being unstable. The most abundant compound in noni juice was 1-hexanol, characterized as having a green aroma, followed by 1-butanol which has a fruity note. Previous analyses of Hawaiian noni fruit volatiles during various ripening and fermentation stages reported 23 prominent volatiles^18^. Of these, 18 were detected in 21-day ripened fruit resembling fermented fruit, and 5 were found in the commercial noni juice used in this study. Notably, 13 of the 28 volatiles in noni juice were alcohols, many of which were absent in the previous analysis. This difference may reflect variations in the fermentation process between laboratory and commercial settings.

Pineapple juice was characterized by 15 volatiles, all esters, 8 of which matched our previous analysis^12^. The most abundant compound in pineapple juice was ethyl acetate, which has a solvent-like fruity aroma. In the juice blends, the detected volatiles were a mix of those found in either noni juice or pineapple juice and their abundance varied based on the proportions of the juices in the blends. For example, ethyl acetate (solvent-like, fruity) was highest in pineapple juice but decreased in blends containing 90% and 80% pineapple juice.

The results show that the rotary evaporation process effectively removed the volatile compounds responsible for the pungent and unpleasant odor of noni juice, as evidenced by the loss of these compounds in the rotary evaporated noni juice and their presence in the water distillate. Notable compounds such as hexanoic acid and octanoic acid, which contribute to the undesirable odor^8,19,20^were efficiently transferred to the water fraction. Also, the rotary evaporation process didn’t significantly alter the color of the juice, maintaining a consistency that was important for sensory evaluation.

Multiple deodorization techniques, including physical, chemical, spice extracts, and distillation methods, have been applied to remove unpleasant odors from various foods. Filteration methods using activated carbon, maltosyl cyclodextrin, and apple polyphenol, have been used to deodorize sweet potato juice^21^. Spice extracts have been effective in reducing the fishy odor of silver carp^22^. However, the deodorization of grated turmeric using a rotary evaporator proved unviable because frequent water additions disrupted the process with initial sensory evaluation showing undesirable flavor change^23^. This suggests that deodorization using a rotary evaporator may be food matrix dependent and not effective for all food types.

### Principal components analysis (PCA)

**Fig. 3:**
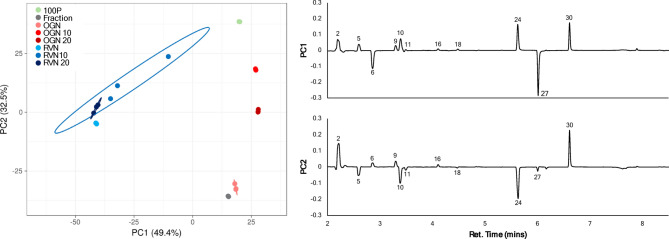
Standardized TIC response PCA with loading plots. Numbered peaks correspond to compounds in Table 2. 95% confidence level is indicated by circles.

**Fig. 4:**
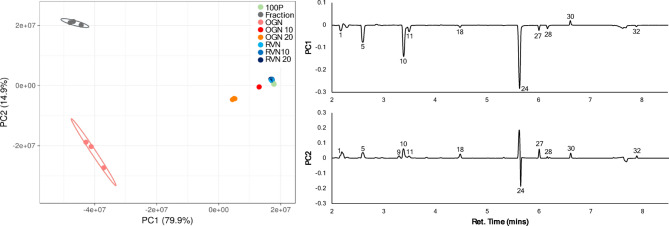
Unstandardized TIC response PCA with loading plots. Numbered peaks correspond to compounds in Table 2. 95% confidence level is indicated by circles.

PCA based on GC-MS TIC values effectively separated juices and showed groupings with “smellier” treatments more closely associated (OGN and Fraction) and other treatments forming a gradient toward “less smelly” (pure pineapple and pineapple plus rotovap noni juice; Figs. 3 and 4). Interestingly, the PCAs are not identical with the standardized PCA reflecting differences in volatile profiles while the unstandardized PCA separated groups based largely on amount of volatiles collected (OGN and Fraction having the highest amounts of volatiles detected).

The standardized PCA evaluating the volatile ‘profiles’ of the juices explains 81.9% of the variation in the first two components, with PC1 accounting for 49.4% and PC2 for 32.5% (Fig. 3). The original noni juice, water fraction, pineapple juice, and the two blends with original noni and pineapple juice showed positive loadings on PC1, while the rotary evaporated noni juice and its blends loaded negatively. The original noni juice and water fraction are negatively loaded in PC2, while the rotary evaporated noni juice and the 20 OGN blend are slightly negative in PC2.

In the PC1 loading plot of the standardized PCA (Fig. 3), four compounds: ethyl acetate (2, solvent-like, fruity), 2-methyl-1-buten-4-ol (10, sweet, fruity), 1-hexanol (24, green), and methyl hexanoate (30, fruit, fresh, sweet), drive the separation on the positive side of PC1 for pineapple juice, original noni juice, the water fraction, and the juice blends with pineapple and original noni juice. For negative loadings, the variation is driven by compounds 2-pentanone (6, acetone like) and 2-heptanone (27, soap, fruity), which separate the rotary evaporated noni juice and its blends to the negative side of PC1. For the PC2 loading plot, ethyl acetate (2, solvent-like, fruity) and methyl hexanoate (30, fruity, fresh, sweet) are the most prominent compounds driving variation on the positive side of PC2. On the negative side of PC2, compounds 1-butanol (5, fruity), 2-methyl-1-buten-4-ol (10, sweet, fruity), and 1-hexanol (24, green), all alcohols, drive the separation of original noni juice and the water fraction.

Examining the unstandardized PCA (Fig. 4), which reflects separations largely based on the relative abundances of each compound, the first two principal components captured 94.8% of the variation: PC1 explains 79.9% and PC2 explains 14.9% of the variation. The fact that PC1 accounts for most of the variation and all but one compound (methyl hexanoate (30, fruity, fresh, sweet)) load in the same direction shows that this PCA largely reflects the amount of volatiles collected which obscures variations in volatile profiles. The original noni juice and the water fraction separate on the negative side of PC1, while pineapple juice and the blends with original noni juice and rotary evaporated noni juice separate on the positive side of PC1. Compounds loading negatively on PC1 include 1-butanol (5, fruity), 2-methyl-1-buten-4-ol (10, sweet, fruity), and 1-hexanol (24, green), which are most abundant in noni juice. 1-hexanol also influences PC2, directing the separation of noni juice to the negative side and the water fraction to the positive side of PC2.

Comparing and grouping complex samples, such as GC analysis with multiple compounds, can be challenging. These analyses are often accomplished using PCA, canonical discriminant analysis (CDA) or a related dimensional reduction technique. In addition, when total ion current (TIC) responses for all time points of a GC-MS profiles are used for PCA, the analysis process is simplified by avoiding individual peak selection, identification, and integrations^16,24^. Another advantage of this approach is the capture of supplementary information from minor peaks and peak shapes that would otherwise be overlooked if peak areas alone were used. Further, several previous studies^16,25,26^ standardized the abundances of detected volatile, thereby creating ‘fingerprint’ profiles for groups of related samples, such as the fruit juices in this study. These unique fingerprint profiles have been found to be strongly dependent on group type (i.e. plant species, food or beverage type, etc.) but relatively independent of the time of collection. The tradeoff with standardizing by run is the loses of data associated with the absolute amounts of particular volatiles present, which is why we run PCA with both the standardized and unstandardized data.

### Sensory evaluation

**Fig. 5:**
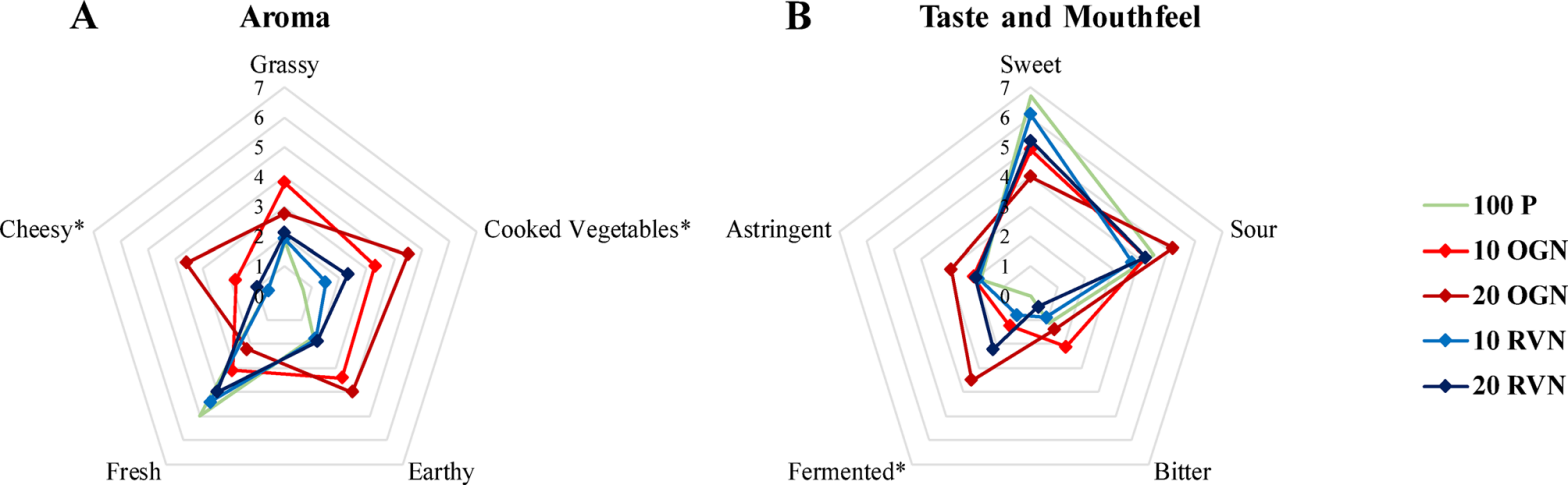
Spider plot representing panelists’ sensory evaluation scores and * shows significant differences (ANOVA, Tukey’s HSD, *p* < 0.05).

The sensory evaluation data indicates that panelists could distinguish between certain attributes in specific samples (Fig. 5). For example, 100% pineapple juice had the highest scores for sweetness and freshness, but these attributes decreased with the addition of noni juice. Notably, the 80% pineapple and 20% original noni juice (20 OGN) had the lowest scores for both sweetness and freshness. Interestingly, the sweetness and freshness of 80% pineapple with 20% rotavap noni juice (20 RVN) scored higher than its counterpart 20 OGN. This suggests that both sweetness and freshness are influenced by the volatiles in noni juice, making the juice more palatable when these volatiles are removed.

While rotary evaporation may remove both unpleasant and pleasant volatile compounds, the overall sensory quality of the resulting blend was improved. This is likely due to the disproportionate impact of pungent volatiles which dominate the aroma profile and mask any pleasant notes. Their removal via rotary evaporation reduced the intensity of the unpleasant odor, even though some fruity volatiles may also have been lost. This aligns with the concept of odor thresholds, where dominant compounds can suppress the perception of others. Sensory testing confirmed that blends made with rotovap-treated noni juice were more acceptable to participants.

Three descriptors: cheesy, cooked vegetables, and fermented significantly differed across the samples. These descriptors were highest in 20 OGN and decreased in 10 OGN. Interestingly, the juices with rotavap noni showed no significant difference in these descriptors compared to 100 P. This indicates that rotary evaporation effectively reduced unpleasant descriptors in noni juice, making it more palatable by removing unpleasant volatiles.

Table 3 shows Pearson correlation coefficients between sensory sweetness, sourness, and related physicochemical properties of Brix/TA, TA, pH, and Brix, with significant correlations denoted by asterisks. Sweetness shows a negative correlation with sourness and pH, but a positive correlation with Brix/TA and Brix. Sourness, on the other hand, is positively correlated with TA and pH. Brix/TA demonstrates a strong positive correlation with Brix and a strong negative correlation with both TA and pH. TA exhibits a positive correlation with pH and a negative correlation with Brix. Lastly, pH negatively correlates with Brix.

**Table 3:** Pearson correlation coefficients between sweetness, sourness, and related physicochemical properties.

	Sweet	Sour	Brix/TA	TA	pH	Brix
Sweet	1	0.31*	0.41	− 0.36	− 0.41	0.43
Sour		1	− 0.34	0.61*	0.43	− 0.06
Brix/TA			1	− 0.9***	− 0.71**	0.92***
TA				1	0.69**	− 0.67**
pH					1	− 0.62*
Brix						1

The *, **, and *** indicate significant differences at *p* < 0.05, *p* < 0.01, and *p* < 0.001, respectively.

## Conclusion

This study refines noni juice, known for its pungent aroma, into a more palatable beverage through rotary evaporation and blending with pineapple juice. By removing volatiles responsible for unpleasant odors, the process improves sensory appeal without altering visual consistency. Blending with pineapple juice further sweetens the taste, potentially producing a beverage for a wider consumer base. The findings show that this method can produce a healthful, marketable beverage using local fruits while supporting sustainable agriculture in Hawaii and preserving the cultural importance of noni.

## Data Availability

The original contributions presented in this study are included in the article. Further inquiries can be directed to the corresponding author through e-mail.
